# Recent Investigations on Tetanus

**Published:** 1892-08

**Authors:** Simon J. J. Harger

**Affiliations:** Professor of Veterinary Anatomy and Zoötechnics, University of Pennsylvania


THE JOURNAL
OF
COMPARATIVE MEDICINE AND
VETERINARY ARCHIVES.

Vol. XIII. AUGUST, 1892. No. 8.

### RECENT INVESTIGATIONS ON TETANUS.

By Simon J. J. Harger, V. M. D.,
*Professor of Veterinary Anatomy and Zoötechnics, University of Pennsylvania.*

Little has been said in American veterinary periodicals concerning the more detailed investigations of tetanus, especially from an experimental standpoint. This has led me to collect and classify into a presentable form some of the most recent information on this subject, trusting that it may be of interest as well as utility to the veterinarian.

The tetanus bacilli are anærobic, and consist, in their vegetative state, of linear rods with rounded ends, isolated or forming chains, and similar but smaller than œdema bacilli ; they show a distinct but feeble inherent mobility, and fructify by means of spherical spores. Stained preparations can be made with the usual analine dyes, and with Gram's method, also with Ziel's double-staining method when they contain spores.

The bacilli are found in earth, manure, compost heaps (Nikolier, Rosenbach, Bonome, etc.), in street dust (Bonome), hay dust (Reitsch), intestinal contents of different herbivora, especially in horse manure (Nocard), wood-splinters, pus from subjects suffering from tetanus, discharge from the scrotum after castration, suppuration in the horse, and in mice after inoculation (Kitt), as well as in the subcutaneous tissue surrounding the seat of inoculation. They usually cohabit with other bacterial forms, from which they can be most readily separated by Kitasato's method, consisting in spreading the pus on blood-serum or agar, and heating it in the culture-oven from 36 to 38 degrees C. ; after forty-eight hours spore-bearing tetanus bacilli are found among their neighbors; this mixed culture is then placed on a water-bath, previously heated to 80 degrees C., for three-quarters of an hour to one hour. The accidental germs are destroyed by this heating process, and only the living spores of the tetanus bacilli survive. An inoculation on gelatine plates, in the presence of moisture, will then develop isolated colonies. According to Villard and Vincent, beef bouillon is used, which, at a temperature of 38 to 39 degrees C., rapidly becomes turbid, and in five to six days shows numerous spore-bearing tetanus bacilli ; this mixed culture is then heated from one to two minutes on a water-bath of 100 degrees C., and spread on plates in an anærobic medium. If this process is not successful the heating may be repeated two or three times.

As to susceptibility, mice, rats, guinea pigs, rabbits, horses, sheep, and pigs (Kitt), will take tetanus by inoculation ; dogs are not very susceptible, but successful inoculation has been observed (Parretti, Kitasato) ; the same is true of cats. Pigeons, likewise, are infected with great difficulty ; one instance has been successful by inoculation from a horse (Kitt, Babes). In sufficient doses to infect the above-mentioned animals, Kitt has been unable to produce tetanus in a cow ; hens also appear to have immunity.

The point of inoculation, under natural conditions, is usually a wound of the integument, which comes in contact with material containing the tetanus bacilli, and is the seat of entrance of the poison (in the horse usually a street nail). In experimental inoculation the infection may be cutaneous, subcutaneous, intra-peritoneal, intra-venous, and sub-dural. The results of FEEDING the infected material have always been negative.

The symptoms of tetanus manifest themselves primarily in the region of the inoculation ; if made at the root of the tail for example, contraction of the muscles of the croup will follow first, then stiffness of the hind members ; from inoculation on the side of the abdomen, pleurosthotonos ; by trepanation of the brain, trismus, spasm of the eye muscles, opisthotonos, and, finally, rapidly acute generalized tetanus. According to the dose and the activity of the culture, tetanus may be acute, subacute, or chronic ; after an incubation of twelve to twenty hours death may follow in fifteen, twenty-four or forty-eight hours. The period of incubation may be from two to eight days, and even three weeks (horse), and after ten to thirty days the disease terminates in death, or the symptoms may abate and the animal recover.

The local symptoms of an inoculation with a pure culture are not marked, excepting a slight hyperæmia, hæmorrhagic spots and slight œdema ; there is no suppuration. The internal organs show blood alterations, hyperæmia, and œdema of the lungs, and, at times, congestion of the mucous membrane, intestinal walls, and glandular organs.

Microscopically, neither bacilli nor spores are found at the point of inoculation, having already disappeared ten hours after the operation (to the staining technique), nevertheless, the bacillus can be reproduced by cultures and the serum of the part is virulent. After a subcutaneous injection or a natural infection, the bacillus can not be demonstrated in any tissue or organ other than the point of inoculation (Kitasato, Villard, Vincent) ; in only one instance, in which nearly the whole brain was used up in making cultures, have Villard and Vincent met the bacillus. The investigators were able to demonstrate the bacillus in large numbers, by means of cultures, in the marrow of bone, the liver, spleen, brain, but never in the blood, after large intra-venous or abdominal inoculations. It appears not to proliferate, as a rule, in the tissues of the body (Kitt). Nevertheless, it is quite well known that the chemical ptomain in the blood and the serous transudations are virulent, and the latter fluids are toxic in mice. Also the blood in the ventricles of the heart and the transudations of persons killed by tetanus contain a toxic substance. This poison exists here in comparatively small proportions, since it requires at least .2 to .3 ccm. to produce tetanus in mice. Bruschettini also found decidedly toxic chemical bodies in the kidneys.

Brieger discovered and isolated poisonous chemical bodies in impure cultures of the tetanus bacillus. Kitasato and Weyl repeated similar investigations, and found toxic bodies (tetanin, tetanotoxin, besides indol, phenol, butyric acid), but concluded that the bacillus produced another principal more toxic than tetanin. Brieger and Frankel afterward believed to have proved that the active poison is a toxalbumen, which was also believed by Tizzoni and Cattini.

Faber, Villard and Vincent state that the akaline fluid, free from germs, obtained by filtering a culture of bacilli through unglazed porous porcelain, still preserves its virulent properties. These scientists furnish extensive information as to the recognition of the characteristics of each toxic agent of tetanus. Kitasato also obtained a germless liquid by filtering the culture through filtering cylinders of unglazed porous porcelain. To be more secure it was filtered twice and placed in a dark chamber. The absence of germs was proved bacteriologically. His inoculations were made with such exactness as to establish an almost uniform minimum dose. In a series of experiments with thirty cultures, five to twenty-one days old, and filtered twice, he obtained the following results: Subcutaneous injections in mice, guinea pigs and rabbits, produced fatal tetanus. The culture filtrate, entirely deprived of bacilli, is so virulent that .0001, .01, .02 ccm. will respectively destroy mice, guinea pigs and rabbits. Injected subcutaneously, the toxic agent enters the blood and produces in these animals tetanic symptoms in five to forty-eight hours, and, at the outside, three days, terminating fatally, sometimes immediately, and at times in five to ten days.

The degree of virulence of a culture is influenced by the duration and the rapidity of the growth of the bacilli in a fresh medium; in old bouillon the growth is slow, and the filtrate is less virulent. In fresh bouillon, with 10 to 15 ccm. of a normal akali solution to one liter, the bacillus proliferates most readily, and the filtrate of such a culture (eight days) is the most poisonous, .000005 to .000008 ccm. killing mice. Heating the filtrate to 65 degrees C. for five minutes, or even less, will destroy its virulence; heating to 60 degrees C., only in twenty minutes; to 55 degrees for one and one-quarter hours has no effect, but after one and one-half hours it becomes inocuous.

Villard and Vincent obtained similar results; they found that the activity of a filtrate becomes greater when used a second time for a bacillus culture. For example, a bouillon culture in which the bacilli have been growing for twenty days gives a filtrate with a minimum fatal dose of $\frac{1}{150}$ ccm. for guinea pigs; when used the second time for a culture medium for eighteen days, the fatal dose will be $\frac{1}{300}$ ccm.

This second filtrate is not nutritious and bacilli will not grow in it; after the addition of a small quantity of fresh bouillon they will again proliferate, and after sixteen days, .001 ccm. of this third filtrate is fatal to guinea pigs. The absolute quantity of the toxic agent in the minimum fatal dose can not be easily ascertained. Kitasato estimated, by drying the filtrate, that .00023 mg. will be fatal to mice, but also remarks that the real fatal quantity is less, because this product also contains ashes and other foreign bodies not peculiar to the tetanic poison. Villard and Vincent estimated the fatal dose for guinea pigs at .000025 g., for mice .00000025 g., 25 mg., thus sufficing to kill about 1,000 guinea pigs and 1,000,000 mice.

The filtrate retains its virulence when dried in a bell jar over sulphuric acid; dried in an ordinary temperature of a room, it partly loses its properties; a temperature of 35 to 37 degrees C., with or without drying, gradually renders it benign (Kitasato). The French investigators showed that the brown amorphous residue of the filtrate dried with sulphuric acid in a room-temperature which retains the odor of the culture, is toxic. Sunlight, which rapidly kills various forms of bacteria and is one of the best natural disinfectants, has the same effect on the virus of tetanus. Kitasato exposed a series of test-tubes to sunlight through a window (temperature 35 degrees—43 degrees C.), and placed a similar series in a dark chamber. The latter preserved their virulence for a long time; one specimen kept in a dark, cold chamber, was after three hundred days as toxic as a fresh filtrate. The former became non-toxic, although it required a long time. Total destruction takes place in fifteen to eighteen hours in direct sunlight; after one-half to several hours small doses (.0001 to .0003 ccm.) otherwise toxic, were found to be without effect. Villard and Vincent obtained similar results from exposure to sunlight and air (thirty-two hours to fourteen days). Dilution with distilled water and bouillon does not alter the toxic properties. The diluted filtrate (1 ccm. to 99 ccm. of water) preserved in a dark chamber is toxic after thirty weeks (Kitasato). This observer investigated the effects of chemicals and blood-serum on the poison. Blood-serum of horses, cattle, rabbits, guinea pigs, and mice is without effect. The same is true of twenty to fifty per centum of ethylproprionat, ethylacetate, methylacetate, amylacetate, iodoform; five per centum of acetate of copper and lead, calomel, cyanide of silver, oleum fagi; 1.5 to 2 per centum of pyrogallol, resorcin, chromic acid, chromate of sodium, branzcatechin, sulphide of sodium, hydrochinon and furfurol; one per cent. of the cyanides of potash and of silver. These chemicals could not be used in larger doses in mice on account of their poisonous effects. The toxic element of tetanus is destroyed in one hour by chloride of iodine, 5 per cent.; cresol, 1 per cent.; sulphuric acid, .735 per cent.; hydrochloric acid, .55 per cent.; phosphoric acid, 1.63 per cent.; oxalic, 1.58 per cent.; acetic acid, 10 per cent.; lactic, 4 per cent.; butyric, 6 per cent.; carbolic, 1.5 per cent.; hydrate of sodium, 3 per cent.; potassium hydrate, .42 per cent.; caustic lime, .1 per cent.; æthylalcohol, 70 per cent.; methylalcohol, 60 per cent. The following chemicals produce the same effects in twenty-four hours; hydrochloric acid, .365 per cent.; nitric, .63 per cent.; sulphuric, .49

per cent.; phosphoric, 1.3 per cent.; oxalic, 1.34 per cent.; acetic, 7.5 per cent.; tartaric and citric, 2.25 per cent.; formic, 1.8 per cent.; lactic, 3.6 per cent.; butyric, 5 per cent.; tannic, 1.5 per cent.; normal alkali solutions oi sodium .3 per cent., and of potassium, .42 per cent.

The general results of Kitasato's experiments indicate that the ptomain of tetanus is sensative to acids, especially the mineral acids, as well as to alkalies, which would be suggested as a dressing for injuries to the foot from which tetanus frequently follows. Corrosive sublimate (five per cent.) is without effect.

The precipitation of the active agent in the filtrate by the addition of chemicals, without altering it, has not been entirely accomplished. Alcohol precipitates it in part, but some remains in solution, of which small doses are fatal to mice. Villard and Vincent found that the poison of tetanus is insoluble in alcohol. The latter substance will dissolve a crystallized mass in a dried filtrate, which is not toxic, while the amorphous residue which remains is slightly soluble in water and is capable of producing death.

These investigators also found that the precipitate with calcium and aluminium phosphate, for example, when thoroughly washed is poisonous without the filtrate losing any of its virulence. This precipitate, exposed to the air, retains its activity for a longer time than the filtrate under similar conditions, which, according to the Frenchmen, remains virulent only for a month.

Ketasato, Villard and Vincent could not accurately determine to which category the tetanic ptomain belongs. It is similar to that of diphtheria (Roux and Yersin) ; also in other respects to the diastatic ferments and snake poison. In the latter comparison it may be mentioned that the germless filtrate will, under certain conditions, liquify gelatine, although such instances are not thoroughly clear, and possibly the filtrate may have contained a diastatic ferment which did the work.

It was for a long time believed that the tetanus bacilli, when introduced into the body through a wound, remain localized, proliferate here, and give off their poisonous agent. There are very few in the wound, and Kitasato has proved by inoculations that they disappear very soon, and are not found here nor in other regions. The same has been observed by many others. Interesting experiments have been made to show that the poison, primarily absorbed by the spores and formed outside of the body in culture fluid, causes the disease, in that the spore-bearing bacilli contain the poison, which is given off to the surrounding tissues (dialysis). It was also found that bacilli, growing for five days at 20 to 22 degrees C., although in large numbers, do not produce toxic effects subcutaneously in guinea pigs in doses of ¼ to ⅓ ccm.; likewise gelatine cultures five to six days old are harmless, inducing the investigators to believe that these young bacilli do not contain sufficient poisonous products, while cultures of ten, fifteen and twenty days gradually increase in their virulence. This is confirmed by the following experiments:

The liquid part of cultures containing only spore-bearing bacilli, heated for twenty minutes at 63 degrees C., lost their virulence without lessening or destroying the vitality of the spores; 1 ccm. of the culture did not cause tetanus. A twenty day old culture of 250 ccm. of bouillon, of which $\frac{1}{400}$ ccm. sufficed to kill guinea pigs in three days, was filtered through porous porcelain, and the residue containing the spores, washed out twenty-four hours afterward, with six liters of water.

The numerous spores, mixed with 4 ccm. of water, and injected into guinea pigs in ½ ccm. doses, did not produce tetanus, yet they retained their vitality and afterward formed virulent cultures. By thorough washing the toxic agent can be washed from the spores which, though otherwise unaltered, will not produce any kind of toxic in the organism. Considering the large number of living spores and spore-bearing bacilli, free from toxic products, that can be injected into as sensitive an animal as the guinea pig without bad effects, while through earth or moisture skin wounds are so easily infected, although only a few bacilli are present, it would seem that there would be some accessory condition in spontaneous infection.

As already shown by Kitasato's experiments, the bacillus will easily produce tetanus after the culture, over five days old, already containing the toxic principal, the bacilli being, so to speak, saturated with it. The virulent products seem to be increased by their association with other processes and other bacterial forms.

Before experimenting with pure cultures it was supposed that the tetanus bacillus only produced its full effects when associated with other bacterial forms; after experimenting with pure cultures which produce fatal results, this idea was negatived, although the possibility of contamination does exist.

Villard and Vincent showed that toxic free tetanus organisms will again become pathogenetic in ¼ to ½ ccm. doses on the addition of lactic acid. Likewise $\frac{1}{15}$ ccm. of a liquid containing spores with a five per cent. solution of lactic injected between the muscles suffices to kill a guinea pig in three days; ⅛ ccm. will destroy a rabbit. Instead of lactic acid, trimethylamine (four drops) can be used for the same purpose.

The French observers have, furthermore, shown that the addition of the micrococcus prodigiosus to the non-virulent spores of the tetanus bacillus favors infection; while if the micrococcus culture be heated or filtered the result is negative. Inoculation with both the normal micrococcus (bouillon culture 5 ccm.) and non-virulent tetanus spores cause the disease in thirty-two hours, and death in fifty hours. Washed micrococci and non-virulent spores cause tetanus, but less quickly, death taking place after six days. It seems that the increased virulence is due to the production of trimethylamine through the m. prodigiosus. In control experiments of injecting the non-virulent spores alone, the subject remained perfectly healthy. The association of different bacteria in relation to pathogenetic effects (Tenth International Medical Congress, 1890, Babes and Cornil) is more or less significant. The general habitat of the bacillus in the earth, dust, etc., and the frequency of traumatisms coming in contact with the latter explain the frequency of tetanus in man, and, according to Villard and Vincent, that other bacteria, or other processes must assist the spores in their activity. This is explained by the following experiment: Toxic free spores were inserted subcutaneously into ten guinea pigs and two rabbits, making an external wound, in five to ten days the subjects became sick, and all died in twenty-four to thirty-six hours. The wound was suppurating and infected with diverse micro-organisms. But not all the accompanying bacteria seem to be able to give virulence to the non-virulent spores. Villard and Vincent's interesting experiments show that the addition of Friedländer's bacilli, the stophylococcus pyogens aureus, and a streptococcus to the non-virulent spores of tetanus, and inserted under the skin with a piece of wood, etc., will not cause tetanus.

The reason that the non-virulent spores do not proliferate in the body, excepting with the addition of lactic acid, micro, predig, etc., producing new poisons, is explained by Villard and Vincent by the consumptive power of the white blood corpuscles. Non-virulent spores, injected alone, are seized and digested by the leucocytes. If the m. prodigiosus and the spores are injected simultaneously, there is an extensive migration of leucocytes which, however, are supposed to consume the micrococci alone, thus giving the spores more time to proliferate and form toxic principles.

Lactic acid diminishes the aggregation of leucocytes ; it plays the part of a repeller (une action vivement repulsive sur les leucocytes), and protects the spores. Toxin-containing spores, even when surrounded by the blood cells, paralyze the latter by their toxic contents and come out victors. Experiments show that spores without toxic properties have a chemico-tactile attraction for leucocytes (demonstrated by Buchner for bacterial protoplasm) and that their toxic contents counteracts this tendency.

These theories are well studied, and to a certain extent true, but phogocytosis in this application has lost much of its support.

*Preventative* innoculations have been practiced in various ways. In July, 1889, Emilio Parietti stated at the Medico-chirurgical Society at Pavia, that dogs that had received small injections of a tetanus culture to produce local tetanic symptoms were protected against large doses sufficient to cause tetanus in those not previously innoculated. Also Guido Tizzoni and Giuseppina Cattani produced immunity in two pigeons inoculated the second time with large subcutaneous doses of a culture.

Only one animal is said to have natural immunity against tetanus—the hen. Inoculation of cows with one-half ccm. of virulent culture has not yet been successful. Kitasato experimented to ascertain if the blood of animals having immunity has any effect upon the production of this condition in other animals. He injected into the abdominal cavity of mice, rabbits, guinea pigs and rats .3 to .5 ccm. of hen's blood and afterward inoculated them with a tetanus culture. The result was negative. He succeeded in obtaining immunity with a pecculiar method, by the use of trichloride of iodine, the substance which Behring used as a preventative for diphtheria. A rabbit received simultaneously, .3 ccm. of a virulent tetanus filtrate and 3 ccm. of a one per cent. solution of the trichloride of iodine ; in twenty-four hours it received .3 ccm. more of the latter solution ; forty-eight hours after the inoculation it became tetanic, the croup and thigh muscles becoming stiff and the back arched. A third injection of the trichloride was given and repeated until five (.15 g.) had been administered. The rabbit was now left alone, the spasms disappeared, and in ten days it had recovered. After fourteen days (from the first inoculation) the same rabbit received 2 ccm of the poisonous filtrate, resulting in slight but transient tetanic symptoms ; after eighteen days, 2 ccm. of a culture, with mild spasms, but more rapid recovery ; after twenty-five days 3 ccm., and later on 5 ccm., without any signs of tetanus. The culture employed was so virulent that a very small quantity sufficed to kill a mouse in thirty hours, and 1 ccm. to kill control rabbits. The trichloride solution did not always produce immunity. Out of fifteen rabbits, Kitasato produced immunity in six, each one receiving a total of 15 ccm. of bouillon culture ; the remaining were not protected, in spite of the generous use of the solution.

The immunity lasted three months. The results were negative in mice and guinea pigs. Other substances, as platinum, gold chloride, creasote, phenal, etc., were without effect. It was possible, with the blood and blood serum of animals with artificial immunity, to produce immunity in rabbits and even to cure those suffering from tetanus, 2 ccm. of the blood or the serum were injected into the peritoneal cavity. After twenty-four hours, these, as well as control mice, were inoculated with tetanus bacilli. The latter died in forty-eight hours from tetanus ; the former remained healthy. The addition of the blood serum of those having immunity to a virulent filtrate, destroyed the virulence of the latter. The immunity, after several inoculations, continue from forty to fifty days. The blood serum of such animals, outside of the body, and preserved in a dark room, retains its preventative properties for one week, then losing it gradually. The blood and serum of rabbits not previously inoculated, as well as that of cows, calves, horses and sheep, has no such properties. The significant experiments of Kitasato and the earlier investigations with trichloride of iodine suggest the importance of making similar experiments in horses suffering from tetanus.

The previous blood inoculations have led Kipp to ascertain whether the blood and serum of horses recovered from tetanus have any power of producing immunity. To this end he inoculated a certain number of mice with the fresh blood-serum obtained by phlebotomy from horses which had tetanus six months and one year before respectively. It was injected under the skin and into the peritoneal cavity in doses as large as ½ ccm All were inoculated from three to ten days afterward with a culture of tetanus bacilli, and all died within a short time from typical tetanus. According to the experiments of Tizzoni and Cattani, it appeared that the blood serum of pigeons and dogs with immunity produced artificially, has anti-toxia properties, ½ ccm. of a virulent culture added to one to two drops, loses its virulence in fifteen to twenty minutes, ½ ccm. of the serum injected into mice under the skin and into the peritoneum protected them against the effects of tetanus cultures. It was without effect in rabbits and guinea pigs ; also when the symptoms of tetanus had appeared before making the injection.

More recent contributions by Tizzoni and Giuseppina Cattani show that the blood serum of animals with immunity obtained with the above method, when uncontamined, kept at a low temperature and in a dark place, will preserve its preventative properties for many days ; it is soon altered by warmth, and partially loses its characteristics at 65 degrees C., and entirely at 68 degrees, if it be exposed for thirty minutes, and at the temperature of coagulating serum. This peculiarity, together with the fact that tetanus anti-toxin is not dialysoble and can be precipitated with alcohol, seem to indicate that the protective substance is albuminous in character, apparently a globulin. These investigations also showed that it is not identical with the fibrin ferment, and that the anti-tetanic properties of the serum are destroyed by hydrochloric acid (one-half drop to five of serum), lactic acid (three drops to five), while an equal part of one-half per cent. solution potassium hydrate preserves it. Ammonium sulphate and magnesium sulphate produces a precipitate, which gives anti-toxic properties to fresh serum. While the blood, as well as the serum, have anti-tetanic properties, the clot after pouring off the serum watery extracts of the muscles, liver and spleen of these animals have sometimes mild and sometimes no such properties.

The precipitated tetanus anti-toxin does not prevent nor cure tetanus in rabits ; in white rats, 2 to 3 cg., of the dried anti-toxin, dissolved in water and injected into the peritoneal cavity, protected the animal against six drops of a virulent culture, the immunity disappearing after five or six days, but could not cure tetanus.

Kitasato also attempted to produce immunity in mice and rabbits with the culture filtrate, with negative results. The mice which had received very small doses, showed symptoms of tetanus, but recovered in about a week, though the injection was only repeated weekly for eight or ten weeks in increasing doses, from .00005 to .0001 ccm. ; subsequently death was produced by .0003 ccm. of the filtrate. Rabbits which survived eight doses .005 to .05 ccm., succumbed when it reached .1 ccm. Mice inoculated with a filtrate weakened by heating, had no immunity. Kitt inoculated five mice with a culture containing spores, producing tetanus in the hind quarters, which disappeared in two to three weeks. After a thorough recovery, these mice were again inoculated with a similar culture, and died in twenty-four to forty-eight hours. In mice, as well as in a few cases in the horse, preventative inoculation has not been protective against tetanus ; in dogs and pigeons, as before mentioned, it has been protective.

Peyraud claims to have attained immunity in rabbits by repeated innoculation with strychnia. Nocard was unable to verify this.

These experiments are, at the present time, still somewhat abstract and conflicting. Nevertheless, it will be an interesting and pertinent work to continue them and ascertain their practical value in relation to tetanus in the higher animals.

